# Novel and emerging physical treatments for major depressive disorder

**DOI:** 10.1016/j.fmre.2025.09.003

**Published:** 2025-09-08

**Authors:** Yang Cai, Dan Wang, Wendi Yang, Siyuan Jiang, Zhiyuan Qiang, Jie Gao, Ling Shen, Honghong Yao

**Affiliations:** a Department of Pharmacology, Jiangsu Provincial Key Laboratory of Critical Care Medicine, School of Medicine, Southeast University, Nanjing 210009, China; b Department of Psychiatry and Psychosomatics, Zhongda Hospital, School of Medicine, Jiangsu Provincial Key Laboratory of Brain Science and Medicine, Southeast University, Nanjing 210009, China; c Co-innovation Center of Neuroregeneration, Nantong University, Nantong 226001, China; d Institute of Life Sciences, Key Laboratory of Developmental Genes and Human Disease, Southeast University, Nanjing 210096, China; e Center for Global Health, School of Public Health, Nanjing Medical University, Nanjing 211166, China

**Keywords:** Depression, Physiotherapy, Music therapy, Light therapy, Cold therapy, Brain stimulation, Future therapy, Central nervous system

## Abstract

Major depressive disorder (MDD), a widespread psychiatric disease with significant impacts on neurological functioning and quality of life, affects 4.4% of the global population. Despite the availability of various treatments, including antidepressants and cognitive behavioral therapy, approximately 50% of patients with MDD exhibit inadequate responses, leading to treatment-resistant depression (TRD). This review evaluates novel physical treatments for depression, focusing on music therapy, light therapy, cold therapy, and brain stimulation techniques such as repetitive transcranial magnetic stimulation (rTMS), transcranial direct current stimulation (tDCS), and deep-brain stimulation (DBS). Music therapy leverages the emotional and social benefits of music to improve mood and cognitive function. Light therapy influences circadian rhythms and neurotransmitter modulation to reduce depressive symptoms. Cold therapy, by regulating the hypothalamic–pituitary–adrenal (HPA) axis and neurotransmitter systems, offers a promising approach for depression management. Brain stimulation techniques, including rTMS, tDCS, and DBS, provide non-pharmacological alternatives by modulating brain activity and connectivity. Although these novel treatments show potential, significant variabilities in clinical outcomes highlight the need for personalized treatment strategies. Future research should prioritize elucidating the mechanisms of these therapies, optimizing treatment protocols, and conducting larger randomized controlled trials to evaluate their efficacy and safety. The integration of advanced technologies and comprehensive mechanistic analyses will be crucial for advancing the field and improving treatment outcomes for MDD.

## 1 Introduction

Major depressive disorder (MDD), a highly prevalent psychiatric condition characterized by substantial disruptions in neurobiological processes and marked impairments in overall quality of life, impacts an estimated 4.4% of the global population [1,2]. Epidemiological data indicate that between 2007 and 2017, the worldwide prevalence of MDD demonstrated a significant epidemiological escalation, with the incidence increasing by approximately 13.0% over a 10-year period [3]. Furthermore, annual relapse rates for MDD are higher than for other psychiatric disorders [4]. MDD negatively affects educational attainment, interpersonal relationships, and occupational outcomes and is associated with increased risks of obesity, cardiovascular disease, and premature mortality [[5], [6], [7]]. The economic burden of MDD is substantial, driven by low productivity and loss of labor h. The impact of MDD on functionality is particularly evident in adults, in whom the coexistence of physical health issues complicates possible treatment options.

The core symptomatology of MDD is defined by persistent depressive affect, anhedonia, and diminished volitional drive. The manifestation of somatization symptoms may occur in a concurrent manner, with the progression of the illness displaying significant variability (i.e., single, recurrent, or chronic). This variability determines the severity of the depression [8,9]. Therefore, MDD is a clinically heterogeneous syndrome characterized by diverse symptom profiles and variable treatment responses. Overall, 30–50% of patients exhibit insufficient responses to first-line treatments, which usually consist of antidepressant medication and cognitive behavioral therapy [10]. Traditional antidepressant medications primarily modulate the neurotransmitter systems implicated in MDD pathophysiology, particularly the serotonin, norepinephrine, and dopamine pathways [11,12]. Effective antidepressants enhance the synaptic and structural plasticity of neurons and regulate monoamines such as serotonin, noradrenaline, and dopamine [13]. However, the therapeutic efficacies of these medications vary significantly. A recent large-scale meta-analysis demonstrated that antidepressants generally exhibited higher efficacy in comparison to placebo, although the overall effect size was not significant [14]. Despite their efficacy, antidepressants are ineffective for one-third to one-half of MDD patients, with many achieving only partial relief. Those unresponsive to two rounds of antidepressants (administered at sufficient doses for at least 4–6 weeks) are typically labeled as having treatment-resistant depression (TRD) [14,15]. The therapeutic response to conventional antidepressants typically requires a minimum of 4 weeks, during which patients frequently experience adverse effects, including sexual dysfunction, diminished libido, gastrointestinal disturbances, headaches, and anxiety. Despite the approval of esketamine by the United States Food and Drug Administration (FDA) for the treatment of TRD, the need to increase the therapeutic armamentarium for depression remains high [16].

**Fig. 1:**
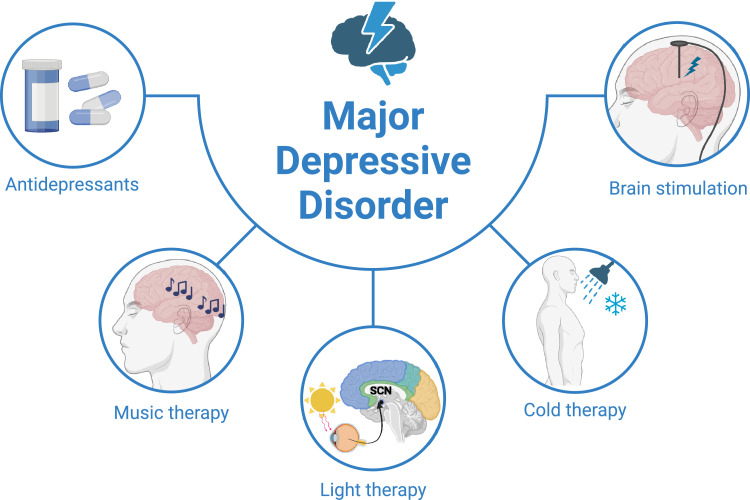
**Therapies for depression**. MDD patients who do not respond adequately to antidepressants can choose physical therapy, including music therapy, light therapy, cold therapy, and brain stimulation techniques. Figure created with BioRender.com.

Given these challenges, it is imperative to investigate and evaluate the efficacies of novel therapeutic interventions that demonstrate a rapid onset of action, improved tolerability, and potentially superior effectiveness compared with existing antidepressants, particularly for TRD patients. This review aims to evaluate novel physical treatments systematically (Fig. 1), highlighting the best available evidence and outlining how these new treatments may be clinically beneficial.

## 2 Music therapy

Music is an effective medium for eliciting and regulating emotional states and moods and is often used intentionally to manage emotions in daily life [[17], [18], [19]]. Music interventions aim to reduce stress, improve mood, and promote self-expression through musical engagement [20]. The healthcare field has acknowledged the effectiveness of music as an evidence-based therapeutic approach for managing depression [21,22]. Juslin indicates that music has the potential to impact motivation, self-perception, and strategies for dealing with challenging emotional states. In certain music therapy approaches, therapists actively assist individuals in processing emotions that have been evoked by music [20]. Music interventions can include various modalities, such as listening, singing, playing instruments, or composing [23]. Evidence indicates that music-based interventions can ameliorate symptoms and enhance functional outcomes in individuals with severe mental illnesses, such as schizophrenia, MDD, cognitive impairments, and substance use disorders [24,25]. As the language of emotion, music aids in understanding, expressing, and altering emotions and is thus a beneficial therapeutic tool [21,26]. Engaging in music-making as a social activity fosters the development of social and communication competencies, thereby enhancing interpersonal relationships [27,28]. Personalized playlists tailored to individual preferences (e.g., self-selected emotionally resonant tracks) demonstrate better efficacy in stress and mood management compared with standardized genres such as classical or ambient music [29,30]. Tempo and rhythmic structure also critically influence outcomes, whereas irregular rhythms may destabilize mood in sensitive populations.

Neurobiologically, sound is processed in the brain through pathways that engage regions involved in emotional regulation, particularly the prefrontal cortex and amygdala [31]. Research has demonstrated that music can provoke profound emotional reactions by activating key brain regions involved in reward processing, motivation, emotional regulation, and arousal, including the orbitofrontal cortex, anterior cingulate cortex, ventral striatum, midbrain, insula, and thalamus [32,33]. These brain structures respond actively to stimuli that induce pleasure and euphoria [34]. In addition, music processing extends beyond the primary auditory cortex, engaging a wide array of neural networks that encompass regions associated with motor functions, emotional reactions, and cognitive processes [35]. Thus, the behavioral effects of music unrelated to the music itself may stem from its extensive connections to neural networks throughout the cerebral hemispheres. Recent studies have shown that music therapy can facilitate emotional processing and modulate stress responses [36,37]. The therapeutic effects of music as a treatment are attributed to mechanisms such as entrainment, the synchronization of brainwave activity with external rhythmic auditory stimuli, and the promotion of relaxation through decreased sympathetic arousal and increased parasympathetic response [38]. A previous study reported that improvisational music therapy combined with standard care was effective in treating adults with depression [39]. Another study demonstrated that group singing significantly attenuated stress and physiological arousal, as evidenced by reduced adrenocorticotropic hormone levels among the participants [40]. These studies indicate that music therapy modulates brain networks involved in emotional processing; however, the precise mechanisms underlying these effects remain to be elucidated. A functional neuroimaging study revealed that music perception engages emotion-related brain networks and can modulate activity in limbic and paralimbic structures, such as the nucleus accumbens (NAcc), hippocampus, hypothalamus, amygdala, insula, cingulate cortex, and orbitofrontal cortex [41]. Dysfunction and structural abnormalities in these regions are characteristic of several psychiatric diseases, including pathological anxiety, depression, and post-traumatic stress disorder [41]. Listening to one’s favorite music induces dopamine release within the striatal system [42], with the caudate nucleus implicated in anticipatory processes and the NAcc associated with reward processing, both of which contribute to the alleviation of negative affect [[43], [44], [45]]. Recent evidence highlighted that music therapy enhances the secretion of neurochemicals critical to emotional regulation—including dopamine, serotonin, and oxytocin—while concurrently stimulating neural networks involved in reward processing and social connectivity, mechanisms that collectively contribute to alleviating depressive symptoms [[46], [47], [48]]. These findings underscore the necessity for further research into the neural mechanisms underlying the therapeutic efficacy of music in addressing these diseases. However, rigorous and high-quality studies are essential to establish robust empirical evidence in support of the therapeutic potential of music [21].

Chronic stress is known to lead to hyperactivation of the hypothalamic–pituitary–adrenal (HPA) axis, which results in sustained increased levels of glucocorticoids (GC, known as cortisol in humans and corticosterone in rodents). High levels of GC can have negative effects on the brain, such as inhibiting neuronal growth and differentiation, leading to atrophy of the hippocampus, and thus affecting emotional regulation [9,49]. A study in mice suggested that music prevents stress-induced depression and anxiety-like behaviors via regulation of the HPA axis. Moreover, listening to music restored serum corticosterone levels, contributing to the restoration of synaptic plasticity and suppression of oxidative stress and inflammation in the hippocampus of chronic stress-treated mice [50]. Although the murine model cannot fully replicate human music perception, both mice and humans can detect music frequencies lower than 20 kHz. In addition, upbeat, slow and heavy metal music (at sound levels below approximately 75 dB) did not elicit aversive or fearful responses in mice [51,52]. These results support the study of the mechanisms by which music influences depressive-like behaviors in mouse models and can help to translate the findings to human applications.

## 3 Light therapy

Alterations in lighting conditions significantly influence diverse physiological and behavioral processes, encompassing circadian rhythms, affective states, and cognitive functions [53,54]. Light therapy has been demonstrated to mitigate depressive symptoms, enhance cognitive performance, and reduce pain perception in humans [[55], [56], [57], [58]]. Clinical studies have established the therapeutic efficacy of light therapy in managing depression [55,[59], [60], [61], [62]], whereas light deprivation has been observed to induce depressive-like behaviors in a variety of species [63,64]. Light therapy offers a non-invasive, cost-effective therapeutic intervention for brain disorders, with minimal side effects and high clinical feasibility. The transmission of light signals via the retina has been demonstrated to exert a significant influence on mood-related behaviors [54]. Elucidating the structural and functional properties of retinal neural circuits is critical for advancing our understanding of the mechanisms through which light modulates depressive-like behaviors.

Light therapy can be effective in improving mood and cognitive function, which may be related to the fact that light therapy affects activity, neurofunctional connectivity, and structural plasticity in multiple brain regions [[65], [66], [67]]. Light therapy functions through the retina, which converts light signals into electrical signals that are then transmitted to the central nervous system [68,69]. The optical rod and cone cells in the retinal nervous system are mainly involved in the visual imaging system as photoreceptor cells; however, intrinsically photosensitive optic ganglion cells (ipRGC) also play an important role in the visual non-imaging system as a third type of photoreceptor cell. ipRGCs not only integrate photic signals from classical photoreceptors but also exhibit intrinsic photosensitivity, enabling direct responses to external light stimuli independent of rod and cone cell input [[70], [71], [72]]. These cells project light information to diverse brain regions, modulating non-image-forming visual functions, including circadian entrainment [53], cognitive processes [73], and affective states [54].

Light therapy mediates its antidepressant effects via photosensitive neural circuits, with the ipRGC–ventral lateral geniculate nucleus and intergeniculate leaflet (vLGN/IGL)–lateral habenula (LHb) pathway identified as critical neural mechanisms underlying its therapeutic efficacy [74]. Light therapy suppresses aberrant neuronal activity in the LHb through the activation of gamma aminobutyric acid (GABA)ergic neurons within the ipRGC-innervated vLGN/IGL, resulting in the amelioration of depressive-like behaviors in mice [74]. Additionally, emerging evidence indicates that the perihabenular nucleus (PHb), situated in the dorsal thalamus, plays a critical role in mediating the adverse effects of light exposure on depressive-like phenotypes. Under irregular photoperiods, neuronal activity in the PHb increases and is accompanied by negative mood changes such as depression and irritability, whereas inactivation of the PHb prevents the mood changes induced by abnormal photocycles. Moreover, it has been reported that the depressive effects induced by rapid circadian photoperiods are mediated through the M1–ipRGC–pHb–mPFC neural pathway [75,76].

The reduced functional activity of monoaminergic neurotransmitters is an important mechanism in the pathogenesis of depression. Light therapy can improve depression by modulating monoamine neurotransmitters, including interfering with neurotransmitter synthesis and reuptake. The rate of 5-hydroxytryptophan (5-HT) release from the brain is positively correlated with light duration and light intensity. Light therapy reduces the function of 5-HT transporters, decreases 5-HT reuptake, and increases the level of 5-HT in the synaptic gap [77]. In addition, light therapy can enhance dopaminergic transmission in most brain regions [[78], [79], [80]].

Light therapy may alleviate depression through its influence on hormonal regulation. Specifically, it modulates melatonin, a circadian rhythm-regulating hormone produced by the pineal gland that promotes sleep. By activating ipRGCs, light therapy inhibits pineal gland activity, thereby reducing daytime melatonin synthesis and secretion, which helps restore circadian homeostasis [[81], [82], [83]]. Morning light exposure stabilizes serotonin transporter (SERT) expression in the prefrontal cortex, sustaining serotoninergic tone throughout the day [84,85]. In addition, light intensity and cortisol levels are closely related; however, the effect of light therapy on cortisol levels is still under debate [86,87]. Consequently, comprehensive empirical studies are required to elucidate the precise impact of light therapy on endocrine regulation.

## 4 Cold therapy

Recent epidemiological evidence has indicated a significant association between the risk of mental disorders and environmental factors, particularly elevated temperatures [88,89]. The neurophysiological mechanisms modulating thermoregulation indicate that environmental temperature may influence mental health outcomes. Given the dual roles of specific neurotransmitters, such as biogenic amines, in modulating both mood and thermoregulation [90], individuals with psychiatric disorders, including depression and schizophrenia, frequently exhibit impaired thermoregulatory function [91,92]. These patients may have difficulty maintaining their body temperature when exposed to drastic temperature fluctuations. In the context of climate change, extreme temperatures are becoming more frequent, intense, and widespread, which may affect individuals’ mental health [[93], [94], [95]]. A recent umbrella review of global evidence that included a meta-analysis of 32 systematic reviews revealed that increased temperature was associated with increases in suicidal behavior and suicide‐ or mental disorder‐related mortality [96]. Extreme heat can cause numerous negative problems, including increased incidence of chronic diseases, negative emotions such as anxiety and stress, increased economic stress and social instability, and changes in lifestyle habits (e.g., sleep patterns), which can lead to worrisome mental health conditions [[97], [98], [99], [100], [101]].

Recent studies have shown a correlation between elevated body temperature and depressive symptoms. A large-scale study involving over 20,000 participants found that individuals exhibiting more severe depressive symptoms consistently presented with higher body temperatures [92]. This suggests that interventions aimed at lowering body temperature might have therapeutic benefits for depression. Notably, cold therapy can influence various physiological processes that are implicated in depression. For instance, cold exposure can regulate the HPA axis, which is often dysregulated in individuals with depression, thereby enhancing adaptation to stress and suppressing pro-inflammatory responses [[102], [103], [104], [105]]. Additionally, cold therapy triggers the release of important hormones and neurotransmitters, including dopamine, serotonin, cortisol, norepinephrine, and β-endorphins, which are integral to mood regulation [106,107]. Several therapeutic approaches involving cold therapy have been explored. One study demonstrated that whole-body hyperthermia, which induces a subsequent cooling effect, was effective in reducing depressive symptoms [108]. This method leverages the body’s natural thermoregulatory responses to achieve therapeutic effects. Another approach involves the use of cold-water immersion or cryotherapy, which has shown promise in improving mood and reducing anxiety [109]. Future research should focus on elucidating the precise mechanisms through which cold therapy exerts its therapeutic effects on depression. Furthermore, large-scale, randomized controlled trials (RCTs) are essential to rigorously validate the efficacy and safety of therapeutic hypothermia interventions. Investigating the potential synergistic effects of cold therapy with existing antidepressant therapies could also provide valuable insights into the efficacy of these treatments.

## 5 Brain stimulation with physical factors

**Fig. 2:**
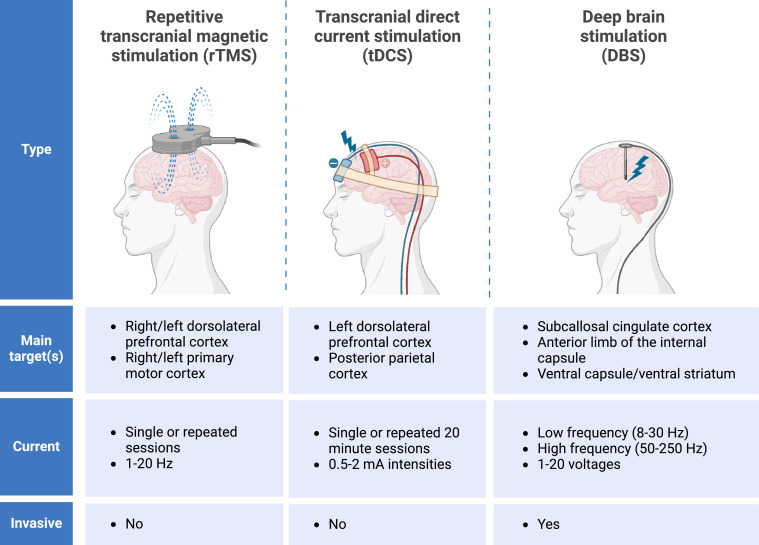
**Brain stimulation techniques and characteristics.** Although the technical parameters of brain stimulation are variable, it is hypothesized that the mechanisms by which the different modalities alleviate depression may be similar. These mechanisms may involve alterations in neural network activity, connectivity, and phase-amplitude coupling, disruption of pathological oscillatory patterns, promotion of synaptic and structural plasticity, as well as regulation of inflammatory mediators. Figure created with BioRender.com.

Brain stimulation therapies, including non-invasive and invasive techniques, have emerged as effective alternatives to pharmacological treatments for depression, offering non-pharmacological approaches to modulate brain function. Several common brain stimulation techniques are depicted in Fig. 2. These methods utilize electrical activity to influence brain connectivity, activity, and oscillatory patterns, although they differ in the intensity and focality of the electric fields applied. Non-invasive brain stimulation (NIBS) techniques, including repetitive transcranial magnetic stimulation (rTMS) and transcranial direct current stimulation (tDCS), selectively modulate cortical regions associated with the pathophysiology of depression. Conversely, deep-brain stimulation (DBS) provides a more invasive but highly precise method to modulate deeper subcortical structures, which are often involved in the neural circuits underlying depression. DBS holds significant promise for treating depression given its ability to modulate activity at any node along a dysfunctional neural circuit with precision, potentially offering a more targeted and effective therapeutic intervention. Regarding brain stimulation therapies (rTMS/tDCS/DBS), current evidence has focused on the modulation of neural network function, with limited direct data available for monoamines. Table 1 presents a classification of invasive therapies (i.e., DBS) and non-invasive therapies (i.e., rTMS and tDCS) in terms of their application and therapeutic efficacy.

**Table 1:** **Invasive vs. non-invasive brain stimulation therapies for MDD**.

Types	Non-invasive therapies		Invasive therapy
	rTMS	tDCS	DBS
Clinical applications	First-line or adjunctive options for moderate MDD, particularly in medication-intolerant patients		Reserved for severe, treatment-resistant MDD after ≥ 4 failed interventions
FDA approval status	Approved for TRD	Investigational use for MDD	
Treatment outcomes	30–40% response rates within 4–6 weeks	20–30% response rates but suitable for home-based protocols	40–60% remission in TRD patients but requires 3–6 months for full efficacy
Risks profiles	• Minimal risks (seizure, transient headache, scalp discomfort) • No surgical injury		• Surgical risks (5–10% infection, 3–5% hardware complications) • Rare neuropsychiatric side effects (e.g., hypomania)
Practical considerations	Outpatient procedures with lower costs and broader accessibility		Requires multidisciplinary teams, long-term follow-up, and significant healthcare costs

### 5.1 rTMS

rTMS is a neuromodulation technique in which focal areas of the cerebral cortex are stimulated with electrical currents generated by magnetic fields. Modern psychiatry considers dysfunctional brain networks to be among the pathological neural bases of depression [110]. rTMS can modulate these dysfunctional networks in a non-invasive manner with minimal side effects [111,112]. rTMS employs a focal magnetic field of sufficient strength (1–2 Tesla) to induce the generation of current in neural tissue, stimulating neuronal signaling [113]. TMS can be used to detect cortical excitability through a single pulse or to induce changes in cortical excitability through repetitive stimulation. rTMS is currently approved by the FDA for the treatment of migraine headaches [114], MDD [[115], [116], [117]], and obsessive-compulsive disorder (OCD) [118]. For MDD, rTMS is used to treat TRD patients, with a remission rate of approximately 20% [119]. However, rTMS is currently rarely individually optimized for stimulation parameters, and few measurements or parameter adjustments are performed during treatment. Therefore, future clinical practice should focus on 1) stratifying patients with different severity levels via pre-measurement and 2) optimizing rTMS by developing personalized treatment parameters.

Neuromodulation functions by dynamically regulating brain networks rather than directly affecting neurotransmitter transmission. The utilization of distinct coil geometries, such as flat, figure-of-eight, and helmet coils, is a critical aspect of rTMS methodology. These coils are designed to emit magnetic pulses at varying frequencies (1–20 Hz) and intensities, modulating the excitability of specific cortical regions [[120], [121], [122]]. Repetitive low-frequency stimulation (1–5 Hz) and continuous θ-pulse stimulation (cTBS) inhibit excitatory synaptic transmission, whereas high-frequency stimulation (5–20 Hz) and intermittent θ-pulse stimulation (iTBS) enhance excitatory synaptic transmission [120,123]. rTMS has been endorsed by the Canadian Network for Mood and Anxiety Treatments (CANMAT) guidelines as an important alternative therapy after antidepressant medication strategies have failed [124,125]. The left dorsolateral prefrontal cortex (DLPFC) and the right prefrontal cortex (PFC) are key targets in rTMS treatment. The left DLPFC, often hypoactive in depression, is linked to poor treatment response, whereas the right PFC may exhibit hyperactivity in MDD. Consequently, high-frequency rTMS is typically applied to the left DLPFC to enhance neural activity, whereas low-frequency rTMS is used on the right PFC to reduce its activity. rTMS is usually stimulated once a day for 30–45 min for 4–6 weeks. As anticipated, the placebo response to rTMS is substantial, although it is notably lower in patients with severe MDD compared with those with mild or moderate depression, independent of sex or age [126]. A 2021 health report that analyzed data from nine systematic reviews and 58 primary studies assessed the efficacy of rTMS versus sham treatment in adults with TRD. The report outlined various rTMS modalities for TRD and concluded that rTMS was an effective intervention. However, it also indicated no significant differences in efficacy across different rTMS modalities [127]. Despite rTMS being an innovative treatment for MDD, the considerable variability in clinical outcomes underscores the necessity for a deeper understanding of MDD pathophysiology and the mechanisms underlying the therapeutic effects of rTMS.

### 5.2 tDCS

tDCS is a non-invasive neuromodulation technique that has shown efficacy in treating MDD, as evidenced in multiple studies [[128], [129], [130]]. In tDCS, a low-intensity current (0.5–2 mA) is applied via scalp-mounted electrodes to regulate the excitability and synaptic plasticity of targeted neuronal populations [131,132]. In contrast to rTMS and electroconvulsive therapy (ECT), tDCS is characterized by its portability, ease of administration, cost-effectiveness, and minimal adverse effects [133,134]. Unlike conventional neurostimulation techniques, tDCS does not elicit direct action potential generation in neurons; rather, it modulates the intrinsic excitability of neural tissue [135]. Importantly, instead of alternating currents that oscillate in direction, the current in tDCS is unidirectional, flowing from the anode to the cathode. The cortical tissue beneath the anode becomes depolarized and more excitable, whereas the tissue beneath the cathode becomes less excitable due to increased polarization of the resting potential. The observed alterations in membrane polarization are insufficient to induce neuronal action potentials directly [136]. These effects endure post-stimulation, with a single session capable of modulating neuronal excitability for periods exceeding 60 min [137,138]. These results indicate that tDCS is linked not only to transient shifts in membrane polarization but also to enduring synaptic plasticity [139]. Moreover, tDCS has been reported to regulate neurotransmitter production.

The standard treatment protocol for tDCS involves 5–10 sessions per day, with a duration of up to 6 weeks. A 2020 meta-analysis of individual patient data from nine studies revealed that tDCS significantly outperformed sham treatment in both response and remission rates, despite a substantial placebo effect [129]. Although tDCS is widely regarded as safe and well-tolerated, rare adverse events, including seizures in individuals with pre-existing epilepsy and skin burns beneath electrodes, have been reported. Furthermore, evidence indicates a 3.3% pooled risk of emergent mania in patients with unipolar depression, with an odds ratio of 5.01 and a risk difference of 0.031 compared with sham treatment [140]. These findings highlight the necessity for longitudinal studies to provide a thorough assessment of the long-term safety profile of tDCS.

### 5.3 DBS

DBS entails the precise placement of electrodes into targeted brain regions using stereotactic techniques. These electrodes are connected to an implanted stimulator device located beneath the skin near the clavicle, which delivers continuous electrical stimulation [141]. DBS offers several advantages over other neurosurgical therapies. First, it is non-ablative and thus reversible, allowing for precise targeting of small, specific brain structures. Second, DBS elicits both acute and sustained therapeutic effects, with adjustable parameters enabling optimization for maximal clinical efficacy [142,143]. Studies have examined the outcomes of stimulating multiple brain regions, such as the subcallosal cingulate cortex (SCC) adjacent white matter, the ventral capsule/ventral striatum (VC/VS), the anterior limb of the internal capsule (ALIC), the median forebrain bundle, and the NAcc [[144], [145], [146], [147]]. However, the majority of these studies lacked controlled designs, making it difficult to exclude potential placebo effects associated with the surgical intervention.

The VC/VS region emerged as a potential target for treating TRD after it was noted that depressive symptoms in patients with OCD improved when they received DBS at this site [148]. Malone *et al*. [149] conducted an open-label trial to evaluate the efficacy of DBS at the VC/VS in reducing depressive symptoms among 15 patients with TRD. At the final follow-up, reductions of 43–45% were observed in both the Hamilton Depression Rating Scale (HDRS) and Montgomery–Asberg Depression Rating Scale (MADRS) scores. The response rate, defined as *a* ≥ 50% reduction in symptom scores, was 53.3%, whereas the remission rates ranged from 33.3% (MADRS) to 40% (HDRS) [149]. Another well-studied target for DBS in TRD is the SCC, a medial prefrontal region situated inferior and posterior to the genu of the corpus callosum. The development of this target has followed a unique trajectory compared with other empirically derived targets. Mayberg *et al*. [150] demonstrated that transient sadness in healthy individuals and depressive symptoms in MDD patients were associated with changes in regional cerebral blood flow within the SCC. Inspired by these findings, researchers administered high-frequency DBS at the SCC in six patients with TRD. In the entire cohort, the HDRS and MADRS scores decreased by 55% and 44%, respectively. Positron emission tomography (PET) imaging revealed normalization of pathological cerebral blood flow patterns following DBS treatment [151]. The adverse effects after DBS can be categorized into surgical (e.g., periorbital edema), somatic (e.g., cephalalgia), and psychiatric (e.g., exacerbation of depressive symptoms or agitation). To date, the application of DBS for patients with MDD remains understudied, necessitating further RCTs incorporating sham controls to rigorously evaluate its therapeutic efficacy [152].

## 6 Regulation of the HPA axis and sympathetic nerves with physical therapy

As described in Section 2, hyperactivation of the HPA axis and subsequent high GC release and sympathetic hyperreactivity are important pathological mechanisms underlying MDD [9,153], which can be targeted and regulated with physical therapy. Music therapy is known to reduce excessive cortisol levels by downregulating activation of the HPA axis [154,155]. Light therapy helps to stabilize circadian rhythms that are closely linked to HPA axis function, thereby restoring circadian fluctuations in cortisol [156,157]. Cold therapy reduces systemic inflammation (e.g., interleukin [IL]-6 and tumor necrosis factor [TNF]-α) [158,159], which is known to exacerbate HPA axis hyperactivation [160]. In addition, it has been reported that cold adaption (e.g., cold water swimming) restores and exercises sympathetic reactivity to increase long-term tolerance to stress [161].

Over the past decades, numerous studies have investigated whether NIBS can regulate HPA axis reactivity in stressful situations [162]. Among eight studies examining prefrontal rTMS effects on cortisol reactivity, four demonstrated consistent modulation of HPA axis activity. Three studies reported that high-frequency rTMS over the left DLPFC—a protocol known to enhance cortical excitability [163]—attenuated stress-induced cortisol release in both healthy individuals [164,165] and patients with bulimic disorders [166]. Conversely, continuous cTBS, which suppresses cortical activity [167], increased cortisol release when applied to the left DLPFC [168]. The pivotal role of the left DLPFC in regulating HPA axis activity has been further supported in tDCS studies. Of the four tDCS studies demonstrating cortisol reactivity modulation, three employed an anodal–left DLPFC/cathodal–right DLPFC montage, which reduced cortisol responses to acute stress [[169], [170], [171]]. These results align with findings from high-frequency rTMS, where excitatory left DLPFC activation similarly suppresses cortisol release. Notably, anodal tDCS over the left DLPFC mimics the neurophysiological excitatory effects of high-frequency rTMS in the motor cortex [172] and the modulation of sympathetic excitability in the periphery (as reviewed by Messina *et al*.) [173], suggesting a shared mechanism of top-down HPA axis regulation for high-frequency rTMS and anodal tDCS. In contrast, cathodal tDCS (inhibitory stimulation) produced inconsistent results, with Antal *et al*. reporting an increase in cortisol responsiveness using a cathodal–left mPFC/anodal–occipital lobe montage [174], which was similar to the inhibitory effect of continuous cTBS.

## 7 Combination therapy for MDD

The integration of physical treatments with pharmacotherapy and psychotherapy has emerged as a transformative strategy to address the pervasive challenges associated with TRD, where monotherapies often fail to achieve sustained remission. For instance, a clinical trial by Lam *et al*. [62] demonstrated that combining bright light therapy (10,000 lux, 30 min/day) with fluoxetine (20 mg/day) yielded a near-tripling of remission rates (58.6% vs. 19.4%) compared with fluoxetine alone in adults with MDD. Similarly, a clinical study in neuromodulation–psychotherapy integration revealed that high-frequency rTMS paired with psychotherapy increased the remission rate compared with rTMS monotherapy (56% vs. 37%) [175,176]. Despite these advances, challenges remain in optimizing treatment planning (e.g., matching circadian rhythms and timing of phototherapy), biomarker-guided personalized interventions (as biomarkers for MDD are lacking), and scaling up the use of multimodal therapies in resource-limited settings.

## 8 Challenges

The reproducibility of the findings related to the efficacy of these therapies in large-scale, rigorously controlled trials is essential for the clinical translation of novel treatments. However, existing evidence demonstrates considerable variability in clinical outcomes, partly attributable to the inherent heterogeneity of MDD. The broad and subjective diagnostic criteria for MDD translate to significant symptom diversity among individuals with the same diagnosis. Consequently, advancing MDD treatment strategies necessitates subgroup stratification and the prediction of pharmacological responses, guided by replicable and objective neurobiological biomarkers/measures to inform clinical decision-making. The development of precise and objective measurement tools is critical to this endeavor [177]. However, predictive techniques for MDD treatment outcomes remain underdeveloped. A systematic review of 12 prognostic models for MDD recovery or remission revealed that most models exhibited poor predictive accuracy and insufficient external validation [178]. Predictive models using objective diagnostic molecular markers are more promising than those currently designed. For example, our study found that partially circular RNA (a special class of non-coding RNA) can accurately and objectively characterize depression severity and treatment efficacy [179,180].

Another challenge is the evolutionary distance between commonly used animal models and the human brain. Currently, the widely utilized animal models of MDD include chronic unpredictable mild stress, behavioral despair, learned helplessness, chronic social defeat stress, drug withdrawal, and transgenic animal models [181]. The evidence for physical therapies tested in animal models of depression is summarized in Table 2. Despite the rich variety of models available, these models still fail to mimic the heterogeneity and complexity of MDD accurately. Recent research is expanding the variety of model arsenals, including the ability to implant collections of cells similar to those in the human brain (known as organoids) into rodent circuits to control behaviors and the ability to conduct *in vitro* studies of circuit function in the intact post-mortem human brain [182,183]. Crucially, insights gleaned from these human-centric approaches can be reverse-translated into animal models, establishing a bidirectional discovery pipeline in MDD research.

**Table 2:** **Preclinical evidence for physical therapies in animal models of depression**.

Therapy	Animal model	Key findings	Reference
Music therapy	CUMS mice	Depressive-like behaviors ↓ Serum corticosterone levels ↓ Hippocampal glucocorticoid receptor expression ↑ Hippocampal BDNF expression ↑ Hippocampal neurogenesis ↑	[184]
		Depressive-like behaviors ↓ Oxidative stress ↓ Serum and brain inflammation ↓ Hippocampal neurogenesis ↑	[50]
	Ovarian hormone withdrawal mice	Depressive-like behaviors ↓ Oxidative stress ↓ Neuroinflammation ↓ Synaptic integrity ↑	[51]
Light therapy	Space restriction mice, Abelson helper integration site-1 knockout mice	Depressive-like behaviors ↓ ATP biosynthesis in PFC ↑ Mitochondrial complex IV expression and activity in PFC ↑	[185]
	CUMS mice	Depressive-like behaviors ↓ Plasma and hippocampal inflammation ↓ Neuronal apoptosis ↓ Hippocampal synaptic function ↑	[186]
	CRS mice, CORT mice	Depressive-like behaviors ↓ Cognitive deficits ↓ Synaptic dysfunction in the PFC and hippocampus ↓ Microglial activation ↓ Neuroinflammation ↓	[187]
	Aversive stimuli mice	Depressive-like behaviors ↓ Retina–vLGN/IGL–LHb pathway ↑	[74]
rTMS	Autoimmune encephalomyelitis mice	Depressive/anxiety-like behaviors ↓ Hippocampal astrocytes activation ↓ Hippocampal neurotoxic reactive astrocyte related gene expression ↓	[188]
	CUMS mice	Depressive-like behaviors ↓ Abundance of *Cyanobacteria* ↑ and *Actinobacteriota* ↓ Levels of polyunsaturated fatty acids in the Hippocampus and PFC ↑	[189]
		Depressive-like behaviors ↓ Microglial activation ↓ Neuroinflammation ↓ Astrocyte loss ↓	[190]
		Depressive-like behaviors ↓ Neuronal loss and apoptosis ↓ Hippocampal neurogenesis ↑ Synaptic plasticity ↑	[191]
tDCS	Acute restraint stress mice, CORT mice	Immobility time ↓ Expression of c-fos in the ventromedial PFC ↑	[192,193]
DBS	CUMS mice	Depressive-like behaviors ↓ γ-aminobutyric acid level in the VTA ↑ Dopamine neuron activity in the VTA ↑	[194]
	CSDS	Sucrose preference ↑ Body weight ↑ Functional connectivity in the dopaminergic pathway ↑ BDNF expression ↑ Mitochondrial biogenesis ↑ Synapse plasticity ↑	[195]

BDNF, brain-derived neurotrophic factor; CUMS, chronic unpredictable mild stress; CRS, chronic restraint stress; CSDS, chronic social defeat stress; CORT, corticosterone; PFC, prefrontal cortex; VTA, ventral tegmental area.

## 9 Conclusion

Significant strides have been made in the realm of physical therapy, particularly in addressing the treatment challenges associated with MDD. Notably, the advancements in physiotherapy approaches are characterized by their ease of administration, cost-effectiveness, and minimal adverse effects, with improved tolerability being a key factor. Although various innovative forms of physiotherapy hold potential for providing targeted treatment for patients with TRD, specialized trials within this specific patient subgroup are imperative to validate their efficacy and optimize protocols. Clinicians can integrate these therapies into practice through a stepped approach: (1) adjunctive use of light therapy or non-invasive neuromodulation (e.g., rTMS/tDCS) for moderate MDD, particularly in medication-intolerant patients; (2) combined regimens (e.g., morning light therapy with SSRIs and rTMS paired with CBT) for TRD to leverage synergistic mechanisms; and (3) biomarker-guided personalization (e.g., circular RNA guided cold therapy and EEG signatures for rTMS–CBT timing) to enhance precision. Multidisciplinary collaboration–coordinating physiotherapy schedules with pharmacotherapy and psychotherapy–is critical for implementation. This emerging evidence may pave the way for enhanced MDD treatment. However, considering the intricate complexity of the human brain and the distinctive nature of the psychiatric conditions involved, the progress of research in physiotherapy has been relatively slow. Future advancements in the field will necessitate the integration of cutting-edge technologies, such as artificial intelligence and computational neuroscience, along with more comprehensive mechanistic analyses.

## CRediT authorship contribution statement

**Yang Cai:** Writing – review & editing, Writing – original draft, Funding acquisition, Conceptualization. **Dan Wang:** Resources. **Wendi Yang:** Resources. **Siyuan Jiang:** Resources. **Zhiyuan Qiang:** Resources. **Jie Gao:** Resources. **Ling Shen:** Validation, Resources. **Honghong Yao:** Writing – review & editing, Project administration, Funding acquisition.
